# On the statistical interpretation of ultrasound‐based models predicting skeletal muscle fat infiltration

**DOI:** 10.1113/EP093815

**Published:** 2026-04-04

**Authors:** Saul Martin‐Rodriguez

**Affiliations:** ^1^ Department of Laboratory Medicine, Division of Clinical Physiology Karolinska Institutet Stockholm Sweden; ^2^ Unit of Clinical Physiology Karolinska University Hospital Stockholm Sweden

**Keywords:** intramuscular fat, muscle ultrasound, predictive modelling, statistical methods

1

I read with great interest a recent study reporting multivariable ultrasound‐derived equations to estimate skeletal muscle fat fraction validated against magnetic resonance imaging (MRI) (Holsgrove‐West et al., 2026). The integration of ultrasound imaging, Dixon MRI fat fraction and proton magnetic resonance spectroscopy within the same experimental framework represents a valuable methodological effort. In particular, the attempt to translate these measurements into clinically accessible predictors of skeletal muscle composition addresses an important challenge in physiological and clinical research.

The multivariable model proposed by the authors, incorporating subcutaneous fat thickness (SFT), echointensity (EI), muscle thickness and age, showed strong associations with MRI‐derived fat fraction, particularly for the vastus lateralis (*R* ^2^ = 0.91). These findings suggest that ultrasound‐derived parameters may contain meaningful information related to intramuscular fat content. Nevertheless, several aspects of the statistical modelling strategy merit careful consideration when interpreting the predictive capability of the proposed equations.

A first point concerns the strategy used for predictor selection. As described in the statistical methods, simple linear regressions were initially performed between MRI fat fraction and candidate ultrasound variables, and predictors reaching statistical significance were subsequently included in a multivariable regression model. While such approaches have historically been common, extensive methodological literature has highlighted important limitations of univariable screening procedures. Variables that do not appear statistically significant in isolation may nonetheless contribute meaningfully in a multivariable context, particularly when predictors are correlated. Conversely, variables selected solely on the basis of univariable significance may enter the final model due to sample‐specific variation rather than reflecting robust associations. For this reason, contemporary statistical guidance generally recommends evaluating predictors jointly when developing predictive models rather than relying on univariable pre‐selection (Harrell, 2015).

A second consideration relates to the interpretation of explained variance in relatively small samples. The predictive equations were derived from a cohort of 28 participants including several predictors in the regression models. In such settings, regression models may be susceptible to overfitting, whereby the apparent predictive performance partly reflects random variation specific to the analysed dataset. This issue has been widely discussed in biomedical modelling studies, where models developed in small samples frequently produce overly optimistic estimates of explained variance (Babyak, 2004). Moreover, the coefficient of determination in multiple regression tends to increase as additional predictors are incorporated into the model, even when these variables contribute limited independent predictive information (Harrell, 2015). Consequently, high *R* ^2^ values should be interpreted cautiously unless supported by appropriate model validation procedures.

These considerations are closely related to a broader distinction in statistical modelling between explanatory and predictive objectives. Traditional regression analyses are often designed primarily to identify associations between variables, whereas predictive modelling focuses on the ability of a model to generate accurate predictions in new datasets. As highlighted in the statistical literature, these two objectives require different modelling strategies and validation procedures (Shmueli, 2010). In this context, the modelling approach adopted in the present study corresponds largely to a classical explanatory regression framework. Demonstrating predictive capability, however, generally requires additional validation procedures specifically designed to evaluate predictive performance.

Internal validation techniques are therefore widely recommended when developing predictive models. Resampling approaches such as bootstrap validation allow estimation of optimism in model performance and provide more realistic assessments of how well a model is likely to perform in independent samples (Babyak, 2004). In addition, modern modelling strategies frequently employ regularised regression approaches, such as ridge regression, lasso or elastic net, which can improve model stability and reduce overfitting through coefficient shrinkage when several correlated predictors are considered simultaneously (Harrell, 2015).

The authors further decomposed the total explained variance using variance importance measures to estimate the relative contribution of each predictor. While such methods can offer useful descriptive insights, their interpretation may be challenging when predictors are correlated. In the present context, echointensity (EI) and subcutaneous fat thickness (SFT) are not physiologically independent variables, as the ultrasound signal is known to be attenuated by increasing subcutaneous fat layers (Pillen & van Alfen, 2011). When predictors share underlying variance, different decomposition approaches may allocate this shared variance differently across predictors, potentially influencing the apparent ranking of variable importance. Relative importance measures should therefore generally be interpreted as descriptive summaries rather than definitive indicators of causal contribution.

Taken together, these considerations do not detract from the experimental strengths of the study nor from the potential value of ultrasound‐derived measures as indicators of muscle composition. Rather, they suggest that the current modelling framework is best interpreted as exploratory. In this context, the results provide encouraging evidence that ultrasound‐derived variables may be associated with MRI‐derived muscle fat fraction. However, confirmation that ultrasound can reliably predict intramuscular fat infiltration will likely require further evaluation in larger cohorts together with modelling strategies specifically designed for predictive inference.

Clarifying this distinction may help align the strength of the conclusions with the statistical framework used to derive the predictive equations while preserving the valuable contribution of this work to the field.

## AUTHOR CONTRIBUTIONS

Sole author.

## CONFLICT OF INTEREST

None declared.

## FUNDING INFORMATION

None.
